# Learning How to Improve the Treatment of Persecutory Delusions: Using a Principal Trajectories Analysis to Examine Differential Effects of Two Psychological Interventions (Feeling Safe, Befriending) in Distinct Groups of Patients

**DOI:** 10.1093/schbul/sbaf083

**Published:** 2025-06-17

**Authors:** Lucy Jenner, Mollie Payne, Felicity Waite, Helen Beckwith, Rowan Diamond, Louise Isham, Nicola Collett, Richard Emsley, Daniel Freeman

**Affiliations:** Institute of Psychiatry, Psychology and Neuroscience, King’s College London, London, SE5 8AB, United Kingdom; Department of Experimental Psychology, University of Oxford, Oxford, OX2 6GG, United Kingdom; Department of Biostatistics and Health Informatics, Institute of Psychiatry, Psychology and Neuroscience, King’s College London, London, SE5 8AF, United Kingdom; Department of Experimental Psychology, University of Oxford, Oxford, OX2 6GG, United Kingdom; Oxford Health NHS Foundation Trust, Oxford, OX4 4XN, United Kingdom; Department of Experimental Psychology, University of Oxford, Oxford, OX2 6GG, United Kingdom; Department of Experimental Psychology, University of Oxford, Oxford, OX2 6GG, United Kingdom; Oxford Health NHS Foundation Trust, Oxford, OX4 4XN, United Kingdom; Department of Experimental Psychology, University of Oxford, Oxford, OX2 6GG, United Kingdom; Oxford Health NHS Foundation Trust, Oxford, OX4 4XN, United Kingdom; Aneurin Bevan University Health Board, Wales, NP18 3XQ, United Kingdom; Department of Biostatistics and Health Informatics, Institute of Psychiatry, Psychology and Neuroscience, King’s College London, London, SE5 8AF, United Kingdom; Department of Experimental Psychology, University of Oxford, Oxford, OX2 6GG, United Kingdom; Oxford Health NHS Foundation Trust, Oxford, OX4 4XN, United Kingdom

**Keywords:** persecutory, delusions, outcome trajectories, psychosis, cognitive therapy

## Abstract

**Background:**

A theory-driven cognitive therapy (Feeling Safe) has produced much better outcomes for patients with persecutory delusions. There are four distinct response classes: very high delusion conviction with large improvement, very high delusion conviction with no response, high delusion conviction with large improvement, and high delusion conviction with modest improvement. Our objective was to apply principal trajectories analysis, a novel statistical method, to original trial data to estimate whether these groups may have responded differently to a different intervention: befriending.

**Design:**

One hundred and thirty patients with persistent persecutory delusions were randomised to six months of Feeling Safe or befriending. Baseline assessments were used to assign patients allocated to befriending (who did not receive Feeling Safe) into the four Feeling Safe response classes. The treatment effect, including on potential mediators, was then estimated for these classes.

**Results:**

Patients in two treatment response classes (Very high conviction/large improvement, High conviction/large improvement) benefited more from Feeling Safe, patients in one group (Very high conviction/no improvement) benefited more from befriending, and patients in the remaining group (High conviction/moderate improvement) benefited equally from the interventions. Mechanism differences were detected when Feeling Safe was superior to befriending, but not when befriending was superior.

**Conclusions:**

There may be patients with psychosis who benefit more from one type of therapy than another, likely due to different change mechanisms. The application of principal trajectories has generated testable hypotheses and a potential step toward personalised treatment. We recommend an investigation of whether sequential provision of the treatment types could enhance patient outcomes. Keywords: persecutory, delusions, outcome trajectories, psychosis, cognitive therapy.

## Introduction

How can psychological therapies for psychosis be improved? One approach is to develop therapies that target the key psychological mechanisms that maintain psychotic experiences,^1,2^ a method applied successfully to persecutory delusions.^3–5^ Moderation and mediation analyses included in tests of therapies suggest further ways to enhance interventions.^6,7^ A complementary approach is to investigate individual responses to treatment,^8,9^ to identify those who benefit and why. This can inform either where the treatment needs to be improved or—potentially—how to personalise provision. We recently identified four different groups of responders to a highly efficacious theory-driven cognitive therapy (Feeling Safe) for persistent persecutory delusions.^10^ This includes one group of patients, about a quarter, who do not respond to Feeling Safe. This prompted consideration of another route for improving treatment provision: using statistical methods to investigate whether there are groups of patients who might benefit from one psychological intervention more than another and why that may be the case. Specifically, could a different intervention, befriending, better help those who do not respond to Feeling Safe? In this paper, we use principal trajectories analysis^11^ to test data collected in a randomised controlled trial that investigated Feeling Safe against befriending for people with persistent persecutory delusions.^12^

Randomised controlled trials, considered the gold standard for treatment evaluation,^13^ report average individual outcomes.^14,15^ For example, allocation to the Feeling Safe therapy has been shown in a randomised controlled trial to lead to an average benefit for a patient of a large additional reduction in delusion conviction above allocation to a befriending intervention (Cohen’s *d* = 0.9).^12^ However, this obscures individual variability in responses. Latent class trajectory analysis is one method that can be used to evaluate patient outcomes at the individual level.^16,17^ This method can classify patients into broadly homogenous subgroups based on their baseline characteristics and recovery trajectories over time.^18,19^ Longitudinal data are modelled using a mixture model framework to identify sub-groups of individuals with similar responses over time, in this instance, when receiving psychological treatment. This approach has been used to investigate treatment responses for a number of mental health conditions.^20–22^ Latent class trajectory analysis usually identifies one group characterised by a lack of response to treatment and remaining groups that improve to varying degrees and speeds.^23–26^

We recently applied latent class trajectory analysis to Feeling Safe session-by-session therapy data and identified four distinct outcome trajectories in patients^10^ (see Supplementary Materials Figure S1). The Feeling Safe trial was a parallel, single-blind, randomised controlled trial testing Feeling Safe against befriending for patients with persecutory delusions (held with at least 60% conviction for at least the past three months). The severity of the persecutory delusions was assessed at baseline before random assignment to one of the two treatments delivered by the same therapists. The severity of the delusions was reassessed at six months (end of treatment) and at 12 months (follow-up). The primary outcome was degree of conviction in the delusion (0-100%). The main trial outcome data were collected by research assessors masked to group allocation. Degree of conviction was also collected by therapists at each therapy session to monitor treatment progress. In order to find the latent class trajectory model that best classified individuals into a subgroup of treatment response, we conducted a series of multinomial logistic models, optimising for good explanatory power with the fewest predictors. There were three significant predictors of sub-group included in the final model: initial delusion conviction, therapy expectancy, and beliefs about others. Patients with very high conviction in their persecutory delusions at baseline split into two response groups: either little or no improvement (Class 1) or large improvement (Class 2). Patients with high delusion conviction also split into two groups: moderate improvement (Class 3) or large improvement (Class 4).

Befriending was also experienced as a valuable intervention for many patients, and over the course of its provision, persecutory delusions reduced (albeit less than with Feeling Safe).^12^ There is the possibility that befriending may have had a degree of efficacy for patients who did not benefit from Feeling Safe.^27^ Principal trajectories analysis is a novel statistical method, building on latent class trajectory methods^28,29^ and extending principal stratification, that can evaluate differential effects of interventions.^6^ This method can be applied when patients have only received one type of intervention but the intent is to estimate at a sub-group level how they might have responded to a different intervention (or control condition). By using the principal trajectories approach, it is possible to move from a descriptive analysis of response patterns to a more robust causal inference approach where within-class comparisons provide clearer insights into treatment effects. As far as we are aware, this approach has not been applied before, except in the example used to describe the statistical method. Such work could support the development of personalisation of treatment provision. Another frontier in a person-centred approach is combining or sequencing different therapies. Research to explore the potential of using befriending to prepare patients diagnosed with schizophrenia for another intervention has been suggested.^27,30^ For example, patients who did not respond to Feeling Safe may have had better clinical responses if it had been preceded by a period of befriending. Perhaps for this subgroup, a slower pace and less challenging content of therapy could facilitate further the development of trust between the therapist and patient.^31^ This may provide better grounds and openness for this subgroup of patients for the later implementation of specific therapy techniques such as behavioural experiments.

Clinical trials often study mediators of therapeutic change at the group level, both common factors across psychological treatments, such as the therapeutic alliance,^32^ or targeted mechanisms used in specific treatments. In the Feeling Safe trial the mediation analysis evaluated mechanisms of change specifically related to understanding Feeling Safe such as safety-seeking behaviours,^33^ negative self-beliefs^34^ and worry.^35^ The findings indicated that a large proportion of the treatment effect on persecutory delusions was explained by many of the hypothesised mediators.^12^ Feeling Safe is personalised: participants are only offered modules targeting specific causal factors that are relevant to them and they choose which to complete. Therefore, the effects of individual mediators would be reduced. The mediators in Feeling Safe have not been investigated at a subgroup level by the different treatment response trajectories.

There is much more to learn about the treatment of psychosis from an individual trajectories approach. It has the potential to lead to personalization, sequencing, and the improvement of treatments. We set out to investigate several questions from further analysis of the Feeling Safe trial: Is one type of therapy (Feeling Safe or befriending) more beneficial for a subgroup of patients with psychosis than another? Can we predict who will benefit most from each type of therapy? What are the mechanisms that may underlie changes from different therapies in each subgroup? By leveraging session-by-session data and applying advanced statistical techniques, we sought to generate hypotheses for future research and ideas for potential ways to improve psychological therapies for patients experiencing persecutory delusions.

## Methods

### Participants

Approval for the trial was received from an NHS Research Ethics Committee (South Central—Oxford B Research Ethics Committee; ref 15/SC/0508). Key inclusion criteria for participants were: aged 16 + years old; a primary clinical diagnosis of non-affective psychosis; and experiencing a persistent persecutory delusion (defined by Freeman and Garety (2000),^36^ held for at least three months) that was held with at least 60% conviction. Exclusion criteria for participants were if they: had a current primary diagnosis of substance or personality disorder, were being treated in forensic mental health services, had an organic syndrome or learning disability, were receiving another psychological therapy or had insufficient comprehension of English. The sample size was fixed as the current study was a secondary analysis of data collected during a randomised controlled trial with 130 participants. The exact sample size required for a principal trajectories approach is hard to quantify, as it will depend on multiple interacting factors: number and size of classes, strength and number of baseline predictors of class membership, and magnitude of within-class effects.

### The Interventions: The Feeling Safe Programme and Befriending

In the randomised controlled trial, half the participants (*n* = 64) were randomly assigned to receive the Feeling Safe programme and the other half (*n* = 66) to receive befriending. Both interventions were delivered as individual therapy by the same clinical psychologists weekly over six-months. An average of 19.1 (SD = 6.7; median = 19) Feeling Safe therapy sessions and 16.4 (SD = 5.7; median = 18) befriending therapy sessions were attended by participants in each arm of the trial.

Feeling Safe is a theory-driven cognitive therapy for persecutory delusions.^4^ The methodological approach to the therapy development has been summarized,^3^ the therapeutic style outlined^37^ and a summary of the design principles has been recently described.^5^ After an initial assessment, a menu of relevant treatment modules is presented to participants. There are up to five modules: Getting better sleep; Winning against worry; Boosting self-confidence; Feeling safe with voices; and Finding safety. Participants choose which modules they would like to work on and in which order. Each module is completed over 6-8 weeks, and there is then a further review, and the next module chosen. Usually, three to four modules are completed over six months. Key outcomes are assessed in each session, including the degree of conviction in the persecutory delusion and the target of the module being used at the time of the session. The key mechanistic driver of the therapy is to help the person directly learn that they are safe in order to counteract the threat beliefs underlying the persecutory delusion. This means that many sessions are conducted outside of the patient’s home or clinic for the person to learn that real-world situations are safe for them now. The Feeling Safe treatment differs from first-generation CBT for psychosis in several ways. For example, addressing new therapeutic targets (e.g. sleep difficulties, worry, positive self-beliefs, and an emphasis on evaluating safety), focusing on one highly manualised modular element at a time, and supporting patients to initiate change through frequent contact between sessions via text and telephone calls.

Befriending, called Feeling Safe and Supported, aimed to simulate how a good friend would respond, with clinicians showing empathy and support and sessions focused on non-threatening topics. A protocol used in previous clinical trials was followed^38,39^ and the expectation was that the sessions would encourage re-engagement in activities and a break from patients' fears.^30^ Sometimes, befriending sessions were also conducted outside in the community. Patients were provided with the following rationale: “The goal of Feeling Safe and Supported is to help you feel safer, happier, and doing more of what you want in life. We know that regular time to connect with other people is good for everyone’s wellbeing. You will have regular time being listened to, respected, and talking about everyday topics. This takes our minds off difficulties and helps us to feel better about ourselves. In Feeling Safe and Supported, you will have time to reflect on interests and activities that you enjoy, which can help to increase motivation to do these activities and spark new interests. This all helps us to feel secure, calm, and connected.”

### Feeling Safe Trial Design and Outcomes

All the data collected in the Feeling Safe trial^12^ were available for secondary analysis, as well as therapist-collected session-by-session data.^10^ The data used for the main trial analysis was collected by blinded research assistants at three time points: baseline, six months (post-treatment), and twelve months (follow up). Baseline measures included in the current report were the Psychotic Symptoms Rating Scale - Delusions (PSYRATS)^40^ used to assess conviction in the persecutory delusion, the Credibility/Expectancy Questionnaire^41^ to measure initial perceptions of likely effectiveness of therapy (taken at the end of the first treatment session) and the Brief Core Schema Scale (BCSS)^42^ to assess positive beliefs about others. We also used measures of psychological variables included in the full mediation model in the original analysis. These included assessment of: beliefs about safety (“I generally feel safe around other people’) and vulnerability (“I feel vulnerable’) on 0-100 visual analogue scales, worry (measured by the Penn State Worry Questionnaire PSWQ^43^), sleep (measured by the Insomnia Severity Index^44^), beliefs about self and other people (measured by the BCSS^45^), anomalous experiences (measured by the SPEQ^46^), jumping to conclusions (measured by the beads task^47^), possibility of being mistaken (measured on a 0-100% scale^48^) and safety-seeking behaviours (measured by the Safety Behaviours Questionnaire^49^).

The session-by-session data used are from patients who received Feeling Safe only. Patients rated conviction in their specific persecutory belief (e.g. “My neighbours are trying to physically harm me”) on a scale of 0-100%, where 0% is “Do not believe” and 100% is “Absolutely certain,” when asked by the therapist in each Feeling Safe therapy session. Delusion conviction ratings were not obtained during the provision of befriending.

### Statistical Analysis

Analyses were conducted in the statistical software Stata version 18 (StataCorp). The analysis we conducted is termed the principal trajectories procedure, and the six steps we took are detailed within the explanation that proposes this method.^6^ We have recently reported the conduct of steps 1–4^10^ that resulted in the classification of individuals in the Feeling Safe treatment group into four latent classes and the patterns of outcome trajectories. In summary, steps 1–4 involved using maximum likelihood to optimise a latent class trajectory model with good explanatory power by comparing a series of latent growth mixture models with different constraints and conditions on classes and probabilities. The four class unconditional trajectory classes and conditional class probabilities model was optimal as it had the lowest Bayesian Information Criteria (BIC), highest predicted probabilities, and made clinical and theoretical sense. Using patients’ individual therapy session-by-session conviction data and baseline predictors, this model enabled us to allocate fifty-five patients to their most likely latent class based on estimated posterior probabilities. Nine patients who were missing baseline predictor data were removed.

Building upon this analysis, we report the conduct of steps 5 and 6. First, we assigned every participant who received befriending to one of the four previously identified Feeling Safe response classes. They were assigned to the latent class that the model predicts they would have been in (the highest probability of belonging to) had they been randomised to receive the Feeling Safe programme rather than befriending. Class assignment was determined by the latent class trajectory model and three baseline predictors: conviction, expectancy, and positive beliefs about others. We removed participants who had missing data for any of the three predictors of the latent class.

Each of the four classes in this study consists of participants who received Feeling Safe and participants who received befriending (every participant only received one of the treatments). For step 6, we ran a within-class regression, controlling for baseline delusion conviction, to see if allocation to Feeling Safe or befriending had a significant effect on delusion conviction. This enabled us to estimate the treatment effect on delusion conviction, the primary outcome, within each class.

Lastly, we investigated potential causal mechanisms of therapy within each class using all the measures from the original Feeling Safe trial mediation analysis (listed in the section above). Using the Analysis of Covariance (ANCOVA) method^11^ we estimated the pathway between treatment and a selection of potential mediators, otherwise known as the *a* path. An observed association within a latent class suggests that the measure of the potential mechanism is influenced by treatment, for the respective class. Given that previous research has established these mediators in the total sample,^12^ we considered confirmation of the *a* pathways to be sufficient and did not explore the full mediation model.

## Results

The 64 patients who were randomized to the Feeling Safe programme remained stratified according to the four latent classes previously reported,^10^ with 55 patients allocated across the four latent classes. Fourteen patients (25%) were allocated to Class 1 (very high conviction, little improvement), 9 (16%) to Class 2 (very high conviction, large improvement), 17 (31%) to Class 3 (high conviction, moderate improvement), and 15 (27%) to Class 4 (High conviction, large improvement). Average predicted probabilities of belonging to each of the four classes were very high, all above 0.9, indicating a high certainty of classification.

Of the 66 patients who were randomized to befriending, 59 were allocated across the four latent trajectory classes. Seven patients were removed from the analysis because of missing baseline predictor data. In total 15 (25%) patients who received befriending were allocated to Class 1, 5 (8%) to Class 2, 27 (46%) to Class 3, and 12 (20%) to Class 4. The average predicted probabilities (Table 1) provide an estimate of certainty in class assignment, and ranged from 0.40 to 0.62: 0.56 for Class 1, 0.40 for Class 2, 0.61 for Class 3, and 0.62 for Class 4. The predicted probabilities of class membership for those who received befriending across the Feeling Safe response classes indicate a satisfactory level of classification certainty for the purposes of this analysis. For Classes 1, 3, and 4, the probability of belonging to the assigned class was at least twice as high as the second most likely class, indicating strong classification certainty. For Class 2, while the probability of belonging to the assigned class is lower (0.40) and therefore less certain, the second-highest probability was 0.29, and no individual within this class had an ambiguous classification.

**Table 1.** Predicted Probabilities of Belonging to Each of the Feeling Safe Responses Classes for People Who Received Befriending.

	Probability of belonging to FS Class 1 (Very high conviction / little improvement)	Probability of belonging to FS Class 2 (Very high conviction/ large improvement)	Probability of belonging to FS Class 3 (High conviction / moderate improvement)	Probability of belonging to FS Class 4 (High conviction / large improvement)
**Class 1 (*n* = 15)**				
Mean	**0.56**	0.25	0.11	0.07
*St.dev*	***0.14***	*0.06*	*0.08*	*0.04*
Min	**0.39**	0.10	0.00	0.01
Max	**0.88**	0.41	0.25	0.13
**Class 2 (*n* = 5)**				
Mean	0.18	**0.40**	0.13	0.29
*St.dev*	*0.12*	***0.02***	*0.07*	*0.06*
Min	0.04	**0.38**	0.08	0.19
Max	0.31	**0.43**	0.22	0.34
**Class 3 (*n* = 27)**				
Mean	0.04	0.11	**0.61**	0.24
*St.dev*	*0.06*	*0.09*	***0.15***	*0.12*
Min	0.00	0.01	**0.34**	0.06
Max	0.22	0.33	**0.88**	0.46
**Class 4 (*n* = 12)**				
Mean	0.01	0.15	0.21	**0.62**
*St.dev*	*0.02*	*0.11*	*0.12*	***0.18***
Min	0.00	0.01	0.06	**0.36**
Max	0.06	0.33	0.43	**0.89**

Descriptive statistics of predictors of latent class at baseline, post-treatment, and follow-up and socio-demographic characteristics are summarized in Table 2. Overall, patients allocated to the Feeling Safe programme or befriending had similar mean predictors at baseline within each class and differed across classes. This indicates the model was successfully allocating people who received befriending to the Feeling Safe response classes according to the three baseline predictors. Socio-demographic characteristics were similar across and within the classes, with differences likely due to chance and small sample sizes.

**Table 2.** Descriptive statistics of predictors and socio-demographic factors across the latent classes.

		Class 1:		Class 2:		Class 3:		Class 4:		Total	
		V high conviction /		V high conviction /		High conviction/		High conviction/			
		Little improvement		Large improvement		Moderate improvement		Large improvement			
		Feeling Safe (*n* = 14)	Befriending (*n* = 15)	Feeling Safe (*n* = 9)	Befriending (*n* = 5)	Feeling Safe (*n* = 17)	Befriending (*n* = 27)	Feeling Safe (*n* = 15)	Befriending (*n* = 12)	Feeling Safe (*n* = 55)	Befriending (*n* = 59)
**Predictors at baseline (pre-treatment)**	Mean conviction (SD)	98.1 (3.7)	98.7 (2.8)	91.3 (9.1)	87.0 (4.5)	83.6 (12.2)	83.9 (12.4)	79.0 (11.1)	75.2 (10.8)	87.3 (12.1)	86.2 (12.8)
	Mean pos. beliefs about others (SD)	4.9 (4.1)	7.4 (3.6)	7.1 (4.8)	4.6 (1.7)	12.2 (3.5)	12.5 (3.2)	10.5 (4.9)	8.7 (2.8)	9.0 (5.1)	9.7 (4.1)
	Mean therapy expectancy (SD)	18.2 (5.5)	19.7 (3.5)	19.3 (4.0)	19.9 (4.2)	16.5 (6.6)	16.3 (4.5)	21.9 (4.1)	21.8 (4.0)	18.9 (5.6)	18.6 (4.7)
**Predictors at 6m (post treatment)**	Mean conviction (SD)	90.9 (18.5)	76.3 (25.2)	17.2 (13.5)	50.0 (26.7)	53.4 (20.8)	54.9 (26.1)	21.4 (28.6)	52.4 (23.5)	48.3 (12.1)	59.7 (26.8)
	Mean pos. beliefs about others (SD)	7.8 (4.4)	8.7 (3.3)	13.2 (5.0)	9.2 (5.2)	11.5 (5.6)	10.6 (4.5)	16.2 (5.7)	10.3 (3.4)	12.3 (6.0)	9.9 (4.1)
**Predictors at 12m (follow up)**	Mean conviction (SD)	82.7 (32.5)	63.3 (37.0)	26.3 (31.9)	60.0 (30.6)	57.0 (23.5)	60.4 (32.5)	26.3 (33.0)	42.5 (28.6)	48.5 (36.9)	58.0 (32.9)
	Mean pos. beliefs about others (SD)	6.8 (3.9)	8.5 (2.8)	12.8 (4.4)	7.8 (4.8)	11.4 (5.0)	10.4 (4.7)	13.0 (4.9)	12.5 (3.6)	11.3 (5.1)	10.1 (4.3)
**Mean age (SD)**		47.9 (10.0)	45.3 (11.1)	38.8 (14.6)	46.6 (6.0)	39.8 (13.9)	41.3 (13.2)	37.4 (9.5)	38.4 (12.2)	41.0 (12.4)	42.2 (12.1)
**Gender (*n*)**	Male	6 (42.9%)	12 (80.0%)	4 (44.4%)	3 (60.0%)	11 (64.7%)	17 (63.0%)	9 (60.0%)	7 (58.3%)	30 (54.5%)	39 (66.1%)
	Female	8 (57.1%)	3 (20.0%)	5 (55.6%)	2 (40.0%)	6 (35.3%)	10 (37.0%)	6 (40.0%)	5 (41.7%)	25 (45.5%)	20 (33.9%)
**Ethnicity (*n*)**	White	10 (71.4%)	14 (93.3%)	7 (77.8%)	3 (60.0%)	16 (94.1%)	25 (92.6%)	11 (73.3%)	9 (75.0%)	44 (80.0%)	51 (86.4%)
	Black Caribbean	1 (7.1%)	1 (6.7%)	1 (11.1%)	1 (20.0%)		1 (3.7%)	1 (6.7%)	2 (16.7%)	3 (5.5%)	5 (8.5%)
	Black African			1 (11.1%)		1 (5.9%)				2 (3.6%)	
	Black other	1 (7.1%)								1 (1.8%)	
	Indian	1 (7.1%)					1 (3.7%)	1 (6.7%)		2 (3.6%)	1 (1/7%)
	Pakistani	1 (7.1%)						1 (6.7%)	1 (8.3%)	1 (1.8%)	1 (1/7%)
	Chinese										
	Other				1 (20.0%)			1 (6.7%)		1 (1.8%)	1 (1/7%)
** Marital status (*n*)**	Single	8 (57.1%)	9 (60.0%)	7 (77.8%)	4 (80.0%)	16 (94.1%)	21 (77.8%)	8 (53.3%)	9 (75.0%)	39 (70.9%)	43 (72.9%)
	Cohabiting								1 (8.3%)		1 (1.7%)
	Married	5 (35.7%)	3 (20.0%)	2 (22.2%)		1 (5.9%)	4 (14.8%)	5 (33.3%)	1 (8.3%)	13 (23.6%)	8 (13.6%)
	Divorced	1 (7.1%)	3 (20.0%)		1 (20.0%)		2 (7.4%)	2 (13.3%)	1 (8.3%)	3 (5.5%)	7 (11.9%)
**Employment (*n*)**	Unemployed	9 (64.3%)	9 (60.0%)	7 (77.8%)	5 (100%)	16 (94.1%)	23 (85.2%)	12 (80.0%)	8 (66.7%)	44 (80.0%)	45 (76.3%)
	Other	5 (35.7%)	6 (40.0%)	2 (22.2%)		1 (5.9%)	4 (14.8%)	3 (20.0%)	4 (33.4%)	11 (20.0%)	14 (23.7%)

**Figure 1.**
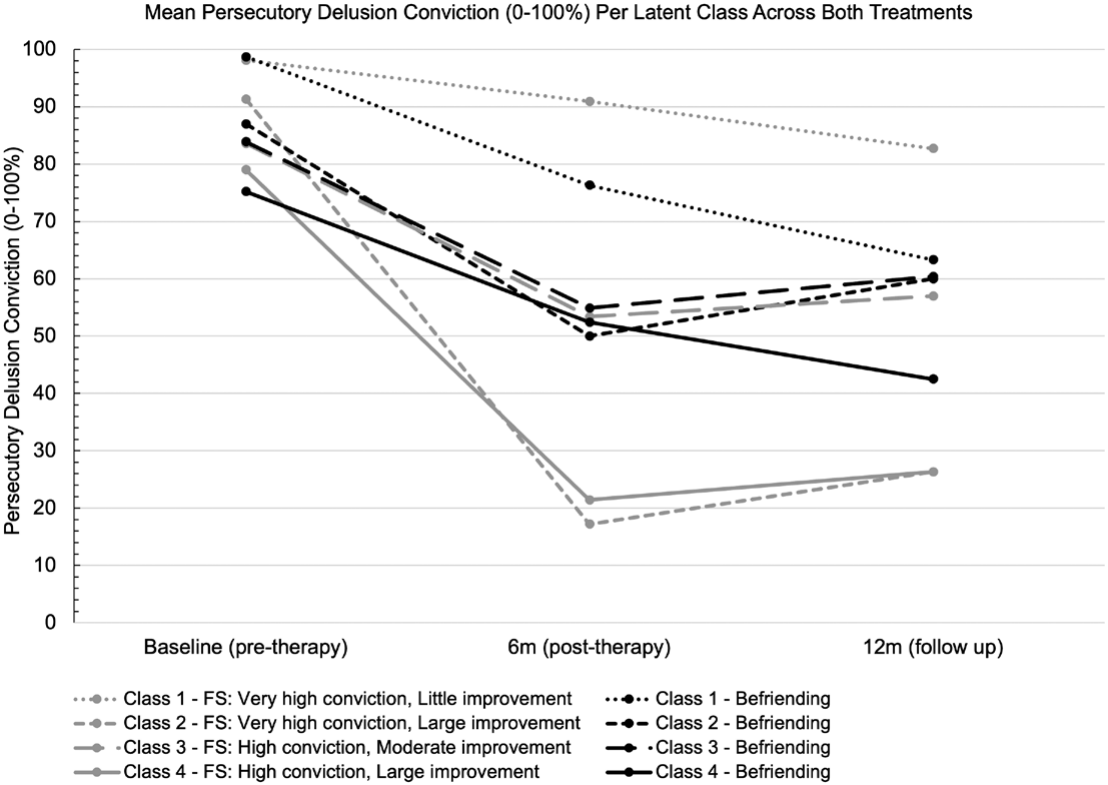
Mean Persecutory Delusion Conviction Per Latent Class Across Both Treatments

The mean persecutory delusion conviction per latent class at baseline, post-treatment, and follow-up across both treatments is also shown in Figure 1. During the course of befriending, the mean persecutory delusion conviction of most of the participants, irrespective of their baseline characteristics, reduced by around 25%. By follow-up, this reduction in persecutory delusion conviction was either maintained or improved further (Classes 1 and 4). The estimated intention-to-treat effects within principal trajectory classes for the Feeling Safe programme compared to befriending at 6 months (post-treatment) and twelve months (follow-up) are shown in Table 3, and the treatment effect for the total sample post-treatment. Overall, the Feeling Safe programme led to significant reductions in persecutory delusion conviction post-treatment compared to befriending in this sample (mean difference = -12.0, *P* = .033). Within Classes 2 and 4, participants allocated to the Feeling Safe programme, post-treatment and at follow-up, reported a reduction in delusion conviction compared to befriending. Participants in Class 1 experienced the opposite; those who received befriending reported a greater reduction in delusion conviction. For Class 3, therapy allocation did not significantly impact change in delusion conviction.

**Table 3.** Intention to treat effects adjusted for baseline persecutory conviction within principal trajectory classes for Feeling Safe compared to befriending at 6 months (post treatment) and 12 months (follow up).

	*n*	Mean difference	Std. err.	95% CI	*P*-value
***Treatment effect total sample (6m)***	*112*	***−12.0***	*5.6*	*-23.1, −1.0*	*0.033*
**Principal trajectory class,** **baseline to post treatment (6m)**					
Class 1 (very high conviction/ little improvement)	29	**16.3**	7.6	0.7, 31.9	0.041
Class 2 (very high conviction/ large improvement)	14	**−32.0**	11.5	−57.2, −6.8	0.017
Class 3 (high conviction/ moderate improvement)	44	**−1.4**	7.4	−16.4, 13.6	0.850
Class 4 (high conviction/ large improvement)	25	**−30.8**	11.3	−54.2, −7.5	0.012
**Principal trajectory class,** **baseline to follow up (12m)**					
Class 1 (very high conviction/ little improvement)	26	**20.0**	13.6	−8.2, 48.3	0.156
Class 2 (very high conviction/ large improvement)	14	**−30.2**	18.7	−71.3, 10.9	0.134
Class 3 (high conviction/ moderate improvement)	43	**−3.0**	9.3	−21.7, 15.8	0.751
Class 4 (high conviction/ large improvement)	24	**−15.5**	13.0	−42.6, 11.5	0.245

### Class 2 and Class 4: Sub-Groups That Benefitted Most from the Feeling Safe Programme

For Classes 2 and 4, controlling for baseline delusion conviction, the Feeling Safe programme reduced delusion conviction compared to befriending. This reduction was statistically significant (*P* < .05) at 6 months (post-treatment) for Classes 2 and 4, reducing threat belief conviction by 32.0 (95% CI = 57.2, −6.8) and 30.8 (95% CI = −54.2, −7.5) points respectively. At 12 months (follow up), the difference in delusion conviction remained lower for people in Class 2 and Class 4 who had received Feeling Safe compared to befriending, by 30.2 percentage points and 15.5 percentage points. The number of patients in each class was small, and the variance at twelve months was greater—almost double for Class 2 (See Table 3). At follow-up, although the reduction in delusion conviction was similar in magnitude to the reduction post-treatment, the differences in outcomes between the Feeling Safe programme and befriending were not statistically significant.

In Class 2, baseline predictors were similar for those who received the Feeling Safe programme and befriending (Table 1). Therapy expectancy and baseline conviction were very similar, and positive beliefs about others were slightly lower in the befriending group compared to the Feeling Safe programme. In Class 4, baseline predictors were comparable, baseline conviction was the lowest, and therapy expectancy was the highest compared to the other three classes.

### Class 1: Sub-Group That Benefitted Most From Befriending

In Class 1, the positive mean difference at 6 months and 12 months of 16.3 percentage points and 20.0 percentage points showed that this sub-group of patients benefited more from befriending than the Feeling Safe programme. This finding was statistically significant at 6 months, but not at 12 months where variance was greater (standard error of 7.6 at 6 months and 13.6 at 12 months).

Patients in Class 1 all had similar baseline predictors, the highest baseline delusion conviction levels across the four groups, and similar therapy expectancy to Class 2, who also had very high baseline conviction levels.

### Class 3: Sub-group Who Benefitted Moderately From Either Treatment

In Class 3, there was no statistically significant difference in delusion conviction between the people who were allocated to befriending compared to the people who were allocated to the Feeling Safe programme at six or twelve months. Both treatments had similar benefits, with delusion conviction reducing by around 25 percentage points to just over 50% conviction post-treatment, and the reduction was maintained at follow-up.

All three baseline predictors were similar in Class 3 across allocation. Compared to the other three classes, patients had the lowest expectations of therapy and higher positive beliefs about others.

### Screening for Potential Mediators

In Classes 2 and 4, where the Feeling Safe programme led to a significantly greater reduction in delusion conviction compared to befriending, evidence suggested potential mechanisms of change. All significant findings are summarized in Table 4. However, no associations were found between treatment and the other potential mechanisms assessed in the trial. No evidence of mechanisms of change was observed in Classes 1 and 3.

**Table 4.** Descriptive statistics and effect of significant putative mediators on treatment outcomes post treatment within latent response trajectory Class 2 (Very high conviction/ large improvement) and Class 4 (High conviction/ large improvement).

Mediator	*n*	Mean (SD)	Mean (SD)	Mean (SD)	Mean (SD)	Mean difference	Std. Err.	95% CI	*P*-value
		**FS (0m)**	**BF (0m)**	**FS (6m)**	**BF (6m)**				
**Class 2**									
Safety belief	14	24.8 (19.3)	38.4 (33.1)	68.9 (23.3)	34.2 (21.1)	27.7	11.7	1.49, 53.70	.040
Vulnerability belief	14	87.9 (14.3)	75.8 (22.8)	40.0 (29.6)	76.2 (19.4)	−40.5	17.1	−78.52, −2.46	.039
Worry	14	66. 2 (6.5)	67.0 (7.1)	44.6 (15.3)	66.4 (10.1)	−21.7	7.0	−37.30, −6.20	.011
Negative beliefs about others	14	15.2 (6.0)	17.6 (6.7)	6.8 (5.0)	15.2 (6.0)	−6.8	2.95	−13.41, −0.28	.043
**Class 4**									
Sleep (insomnia)	25	11.1 (8.3)	16.1 (6.2)	5.3 (4.5)	14.1 (7.6)	−6.8	2.2	−11.46, −2.23	.006
Positive beliefs about others	25	10.5 (4.9)	8.7 (2.8)	16.2 (5.7)	10.3 (3.4)	4.7	2.15	0.14, 9.18	.044

FS = Feeling Safe. BF = Befriending.

Within Class 2, at the end of treatment, those who received the Feeling Safe programme had an average safety belief rating 27 points higher than the befriending group (mean difference = 27.7, 95% CI = 1.49, 53.70) and an average vulnerability score 40 points lower (mean difference = −40.5, 95% CI = −78.52, −2.46). Those in Class 2 who received the Feeling Safe programme also reported worrying less (mean difference = −21.7, 95% CI = −37.3, −6.2) and having reduced negative views of others (mean difference = −6.8, 95% CI = −13.41, −0.28) after treatment.

Within Class 4, at the end of treatment, the Feeling Safe group had a significantly lower insomnia score than the befriending group (mean difference = −6.8, 95%CI = −11.46, −2.23) and significantly higher positive beliefs about others (mean difference = 4.7, 95%CI = 0.14, 9.18).

## Discussion

Even with the best psychological therapy for delusions there is a proportion of patients who do not benefit. We wanted to exploit a unique data set—in which the best theory-driven psychological therapy was compared to a very different therapeutic approach—to generate potential testable ways to improve psychological therapy provision for persecutory delusions. A novel statistical method was applied in order to investigate differential treatment outcomes to psychological therapy within sub-groups of patients with psychosis. The analysis indicated that many patients may respond better to Feeling Safe than befriending. But for the patients with very high delusion conviction who did not respond to Feeling Safe, the findings indicate that these patients may well have done better with befriending. Allocation to therapies based on the baseline assessments is not sensitive enough yet to personalise therapy provision at the individual level, but could become a focus for future research when the therapy is deployed at a greater scale. It also indicates that research is warranted to test whether, for patients with very high levels of delusion conviction, there could be benefits of adding an initial component of befriending style work before Feeling Safe. Broadly, there could be research on the sequencing of therapy approaches. As hoped, a focus on individual responses has generated hypotheses for the development of better therapy, especially for those people who do not currently benefit from the most effective psychological treatment for persecutory delusions. A key limitation of the work is that it is based on secondary statistical modelling of a pre-determined sample from one randomised controlled trial rather than a direct prospective test.

Previously, we identified four latent classes reflecting different outcome trajectories in response to the Feeling Safe programme. In this study, we used baseline predictors to estimate the likely Feeling Safe response trajectory class membership for patients who received befriending (but not Feeling Safe). This allowed us to make informative comparisons across treatment conditions within each latent trajectory class. Overall, these findings show that Feeling Safe can lead to very large reductions in delusion conviction, more than sixty percentage points for most people who respond well to it. Befriending reduces delusion conviction to some extent across the four identified latent classes, ranging from 20 to 37 percentage points on average. However, befriending never successfully reduces mean delusion conviction in a class below 50% after treatment. This is consistent with the main trial report that the odds of a halving of delusion severity were almost eight times greater with Feeling Safe than befriending.^12^ Therefore, befriending may well have a modest but fairly consistent patient benefit, while Feeling Safe often leads to major change, but a proportion of patients do not benefit.

Patients in Class 2 and Class 4 who received Feeling Safe demonstrated the most substantial reductions in delusion conviction compared to those who received befriending. For Class 2 (characterized by very high conviction at baseline), there was a significant mean difference in the reduction of delusion conviction, favoring Feeling Safe by thirty-two percentage points at six months and thirty percentage points at twelve months. Throughout the provision of befriending there was a reduction in delusion conviction in people in Class 2; their mean conviction rating reduced from 87% to 50% at six months. However, for those who received Feeling Safe, the mean conviction rating reduced from 91% to 17%. Similarly, in Class 4 (characterized by high conviction at baseline), there was a mean difference in the reduction in conviction favouring Feeling Safe of 31 percentage points at 6 months and 16 percentage points at 12 months. Similar to Class 2, those who received befriending in Class 4 still benefited from it, with a reduction in mean conviction rating from 75% to 52% after treatment, compared to a reduction from 79% to 21% for those who received Feeling Safe.

In contrast, Class 1 patients, who had very high conviction ratings before treatment across the four groups, derived greater benefit from befriending than from Feeling Safe. At 6 months there was a significant mean difference of 16 percentage points for befriending compared to Feeling Safe. The mean conviction rating reduced from 99% by more than 20 percentage points to 76% with befriending, but for those who received Feeling Safe conviction rating reduced by less than 10 percentage points from 98% to 91%. The mean difference favoring befriending at 12 months was 20 percentage points, with a further reduction in mean conviction rating from 76% to 63% for those who received befriending.

The sample sizes were small. Clearly, replication is needed. But to have identified significant sub-class differences indicates that the findings may be robust. Baseline predictors for Class 1—including very high conviction, low positive beliefs about other people, and comparable therapy expectancy to Class 2—suggest that befriending may provide a more suitable initial approach for a proportion of patients whose strong degree of conviction in the delusion could impede direct engagement with beliefs about harm and safety. Such patients may benefit from a more relationship-focused intervention, like befriending, before transitioning to a structured cognitive therapy. It has been previously suggested by evaluators of befriending that such a phased approach might optimize outcomes for a proportion of people.^27,50^ It could be possible that other therapeutic approaches may also be good complements to Feeling Safe. However, it is not yet possible to reliably allocate individuals to a trajectory group, particularly predicting for people with very high conviction (Classes 1 and 2) whether they will respond better to Feeling Safe or befriending. Reliably predicting the most likely response to different psychological interventions for an individual will require the development of robust allocation models based on considerable research. Our results are an initial indication of a route to be studied.

The participants in Class 3, characterized by high (but not very high) delusion conviction, lower therapy expectations, and relatively higher positive beliefs about others, showed moderate improvements in delusion conviction with both interventions. The conviction reduction of around 25 percentage points was sustained at follow-up in both treatment groups, and therapy type did not significantly affect outcomes. These findings suggest that both therapy approaches delivered by clinical psychologists are comparably effective for some patients, and factors such as patient preference or service considerations might be prioritised to guide treatment selection. It is important to remember, however, that in this study the same skilled therapists (clinical psychologists) delivered both interventions, and considerable efforts were made to engage participants in befriending for a six-month period. The uptake of befriending was very high with these therapists. Befriending delivered by different workforces, for example, untrained volunteers, in different settings, or for a different length of time, may lead to different findings and different health economic considerations.

The analysis of the mediators builds upon findings from the original report of the Feeling Safe trial, which showed that the overall treatment effect of Feeling Safe could be explained by many of the proposed mediators derived from the theoretical model underpinning the work.^51–53^ The current study allowed the exploration of mediation within the sub-group response trajectories. Different mediators were significant within the different latent classes, suggesting the main mechanisms of change could differ across sub-groups. When interpreting these findings, it is important to hold in mind that the modules completed can differ for each person, and these findings could partly reflect the different proportions of patients completing each module within the sub-groups. Within Class 2—people who had very high delusion conviction before treatment, that reduced substantially from Feeling Safe—beliefs about safety and vulnerability, worry, and negative beliefs about others reduced significantly more with Feeling Safe. Within Class 4—people who had high delusion conviction that reduced substantially after Feeling Safe—sleep and holding more positive beliefs about others were significantly improved. The differences observed in mediators highlight the complexity of factors underlying delusional beliefs and underscore the need for flexible, patient-specific treatment components. Recent research points to additional social and cognitive factors to be explored,^54^ such as discrimination^55,56^ or dissociation,^57^ that could be tested for moderation or mediation in future trials of interventions.

The study had several limitations. First, the sample size was small for comparing two treatments, which limits the ability to detect smaller differences, especially given missing data. The resulting subgroups are also small—one latent class had only five participants receiving befriending—requiring caution in interpreting findings. This analysis should therefore be viewed as exploratory, to generate hypotheses for future investigation. Second, the statistical method used depends on identifying strong baseline predictors of outcomes. The study was not originally designed with this analytical approach in mind, and not all factors that could influence outcomes were measured. Although we tested many baseline variables in our earlier work,^10^ only three moderately predictive baseline variables were included in our final model. Our findings remain tentative and could be influenced by other untested factors, such as imagery,^58^ aberrant salience^59^ or dissociation.^54^ As expected, the average posterior probabilities for each class were lower for those who received befriending compared to Feeling Safe, indicating a lower certainty of class assignment. In addition, our mediation analyses were constrained by the available data: the mediators collected were primarily focused on mechanisms relevant to the Feeling Safe intervention. Specific potential mediators of befriending,^27^ such as the therapeutic relationship or participants’ activity levels outside of the sessions, were not examined. Another important limitation is the generalizability of our results. The study was conducted at a single site and included relatively few participants from ethnic minority backgrounds. This homogeneity may reduce the external validity of our findings, highlighting the need for future research in more diverse and multi-site settings.

Importantly, the methodological approach could have value more broadly in intervention research, and our findings suggest avenues for future research that could lead to better outcomes for patients with persecutory delusions in response to psychological interventions. For example, a randomised test of the sequencing of Feeling Safe and (a shorter version) of befriending. Additionally, testing other potential mediators, particularly those more relevant to befriending, could deepen our understanding of mechanisms across interventions. This learning could lead to better versions of the interventions. The whole approach—collection of session-by-session data and use of trajectory analyses—would benefit from greater scale across psychosis services and research trials.

## Supplementary Material

sbaf083_suppl_Supplementary_Figure_S1
