# Supplementary material for: Learning How to Improve the Treatment of Persecutory Delusions: Using a Principal Trajectories Analysis to Examine Differential Effects of Two Psychological Interventions (Feeling Safe, Befriending) in Distinct Groups of Patients
Source: Schizophr Bull. 2025 Jun 17;52(1):sbaf083. doi: 10.1093/schbul/sbaf083 (PMC12809788; doi:10.1093/schbul/sbaf083)
Supplement: sbaf083_suppl_Supplementary_Figure_S1 [file sbaf083_suppl_supplementary_figure_s1.docx]

**Supplementary materials:**

**Improving the treatment of persecutory delusions: learning from differential effects of two psychological interventions in distinct groups of patients**

Authorship: Lucy Jenner [1,3], Mollie Payne [2], Felicity Waite [3,4], Helen Beckwith [3,4], Rowan Diamond [3,4], Louise Isham [3,4], Nicola Collett [5], Richard Emsley [2], Daniel Freeman [3,4]

[1] Institute of Psychiatry, Psychology and Neuroscience, King’s College London, London, UK

[2] Department of Biostatistics and Health Informatics, King’s College London, London, UK

[3] Department of Experimental Psychology, University of Oxford, Oxford, UK

[4] Oxford Health NHS Foundation Trust, Oxford, UK

[5] Aneurin Bevan University Health Board, Wales, UK

Corresponding author: lucy.e.jenner@kcl.ac.uk

**Supplementary materials, Figure 1: Mean persecutory delusion conviction rating per session across patients for each latent trajectory class identified in a previous study**^1^

**Supplementary materials, Stata code used for the 1) latent class trajectory analysis and 2) principal trajectories analysis.**

We have uploaded the Stata code files to github so they are also easily accessible in the correct format: <https://github.com/molliejpayne/Feeling-Safe-Latent-Trajectories>

1. **Latent Class Trajectory Analysis**

The following code was used for the latent class trajectory analysis described in a previous paper^1^ and this analysis is a prerequisite for the principal trajectory analysis.

This script implements a latent trajectory model, which compared the changes

of threat belief over time, for patients who received Feeling Safe therapy. We

explore various models, comparing different class solutions and constraints, and

choose the model that is best fit to the data and makes most theoretical sense.

The best fitting model will have the lowest Bayesian Information Criteria. The

final model will divide our sample into classes, which describe the main

trajectories of change for threat belief over time, conditional on some baseline

predictors.

**Contents of Script:**

- Data Preparation

- Missing Data Summary

- Find Number of Classes

- Define Constraints

- Comparison of Models

- Model 1 - Unconditional Trajectory Classes and Unconditional Class Probabilities Model

- Model 2 - Unconditional Trajectory Classes and Conditional Class Probabilities Model

- Model 3 - Conditional Trajectory Classes and Unconditional Class Probabilities Model

- Final Model Plots

**#---Data Preparation-----------------------------------------------------------

clear

cd "/DataFileLocation" //Set the directory to the location of your file

use FeelingSafeData //Open Data file

**#---Missing Data Summary-------------------------------------------------------

//threat_b_* represents the outcome (threat belief) at each time point.

misstable summarize threat_b_* //Summary of all missing data in outcome

misstable summarize threat_b_*, generate(miss_) //Gen new variable for missingness

misstable pattern threat_b_*, freq bypattern //Missingness pattern

**#---Find Number of Classes-----------------------------------------------------

/* This marks the initial stage of the latent trajectory analysis. In this phase,

we will examine various class solutions, beginning with a single-class solution

(lclass (C 1)). For each model, all time points (1–19) for the outcome variable,

threat_b_, are included. The residual error for the outcome is constrained to be

consistent across all measures of threat belief (e.threat_b_@a).

We then calculate and record the Bayesian Information Criterion (BIC). As the

number of classes increases, the BIC is expected to decrease. Once the BIC begins

to rise, we can stop adding more classes. For models with two or more classes, we

also calculate the posterior probabilities of membership in each class.

The optimal number of classes is determined by identifying the model with the

lowest BIC and the best posterior probabilities.*/

*One Class Solution

gsem (threat_b_1 threat_b_2 threat_b_3 threat_b_4 threat_b_5 threat_b_6 threat_b_7 threat_b_8 threat_b_9 threat_b_10 threat_b_11 threat_b_12 threat_b_13 threat_b_14 threat_b_15 threat_b_16 threat_b_17 threat_b_18 threat_b_19<-), //

lclass (C 1) var(e.threat_b_1@a e.threat_b_2@a e.threat_b_3@a e.threat_b_4@a e.threat_b_5@a e.threat_b_6@a e.threat_b_7@a e.threat_b_8@a e.threat_b_9@a e.threat_b_10@a e.threat_b_11@a e.threat_b_12@a e.threat_b_13@a e.threat_b_14@a e.threat_b_15@a e.threat_b_16@a e.threat_b_17@a e.threat_b_18@a e.threat_b_19@a)

estat ic //AIC = , BIC =

*Two Class Solution

gsem (threat_b_1 threat_b_2 threat_b_3 threat_b_4 threat_b_5 threat_b_6 threat_b_7 threat_b_8 threat_b_9 threat_b_10 threat_b_11 threat_b_12 threat_b_13 threat_b_14 threat_b_15 threat_b_16 threat_b_17 threat_b_18 threat_b_19<-), //

lclass (C 2) var(e.threat_b_1@a e.threat_b_2@a e.threat_b_3@a e.threat_b_4@a e.threat_b_5@a e.threat_b_6@a e.threat_b_7@a e.threat_b_8@a e.threat_b_9@a e.threat_b_10@a e.threat_b_11@a e.threat_b_12@a e.threat_b_13@a e.threat_b_14@a e.threat_b_15@a e.threat_b_16@a e.threat_b_17@a e.threat_b_18@a e.threat_b_19@a)

estat ic //AIC = , BIC =

*Calculate Posterior Probabilities

predict pr2_*, classposteriorpr

egen maxprob2 = rowmax (pr2_1 pr2_2)

gen class2 = 1 if pr2_1 == maxprob2

replace class2 = 2 if pr2_2 == maxprob2

bysort class2: summ pr2_*

*Three Class Solution

gsem (threat_b_1 threat_b_2 threat_b_3 threat_b_4 threat_b_5 threat_b_6 threat_b_7 threat_b_8 threat_b_9 threat_b_10 threat_b_11 threat_b_12 threat_b_13 threat_b_14 threat_b_15 threat_b_16 threat_b_17 threat_b_18 threat_b_19<-), //

lclass (C 3)var(e.threat_b_1@a e.threat_b_2@a e.threat_b_3@a e.threat_b_4@a e.threat_b_5@a e.threat_b_6@a e.threat_b_7@a e.threat_b_8@a e.threat_b_9@a e.threat_b_10@a e.threat_b_11@a e.threat_b_12@a e.threat_b_13@a e.threat_b_14@a e.threat_b_15@a e.threat_b_16@a e.threat_b_17@a e.threat_b_18@a e.threat_b_19@a)

estat ic //AIC = , BIC =

*Calculate Posterior Probabilities

predict pr3_*, classposteriorpr

egen maxprob3 = rowmax (pr3_1 pr3_2 pr3_3)

gen class3 = 1 if pr3_1 == maxprob3

replace class3 = 2 if pr3_2 == maxprob3

replace class3 = 3 if pr3_3 == maxprob3

bysort class3: summ pr3_*

*Four Class Solution

gsem (threat_b_1 threat_b_2 threat_b_3 threat_b_4 threat_b_5 threat_b_6 threat_b_7 threat_b_8 threat_b_9 threat_b_10 threat_b_11 threat_b_12 threat_b_13 threat_b_14 threat_b_15 threat_b_16 threat_b_17 threat_b_18 threat_b_19<-), //

lclass (C 4)var(e.threat_b_1@a e.threat_b_2@a e.threat_b_3@a e.threat_b_4@a e.threat_b_5@a e.threat_b_6@a e.threat_b_7@a e.threat_b_8@a e.threat_b_9@a e.threat_b_10@a e.threat_b_11@a e.threat_b_12@a e.threat_b_13@a e.threat_b_14@a e.threat_b_15@a e.threat_b_16@a e.threat_b_17@a e.threat_b_18@a e.threat_b_19@a)

estat ic //AIC = , BIC =

*Calculate Posterior Probabilities

predict pr4_*, classposteriorpr

egen maxprob4 = rowmax (pr4_1 pr4_2 pr4_3 pr4_4)

gen class4 = 1 if pr4_1 == maxprob4

replace class4 = 2 if pr4_2 == maxprob4

replace class4 = 3 if pr4_3 == maxprob4

replace class4 = 4 if pr4_4 == maxprob4

bysort class4: summ pr4_*

*Five Class Solution

gsem (threat_b_1 threat_b_2 threat_b_3 threat_b_4 threat_b_5 threat_b_6 threat_b_7 threat_b_8 threat_b_9 threat_b_10 threat_b_11 threat_b_12 threat_b_13 threat_b_14 threat_b_15 threat_b_16 threat_b_17 threat_b_18 threat_b_19<-),//

lclass (C 5)var(e.threat_b_1@a e.threat_b_2@a e.threat_b_3@a e.threat_b_4@a e.threat_b_5@a e.threat_b_6@a e.threat_b_7@a e.threat_b_8@a e.threat_b_9@a e.threat_b_10@a e.threat_b_11@a e.threat_b_12@a e.threat_b_13@a e.threat_b_14@a e.threat_b_15@a e.threat_b_16@a e.threat_b_17@a e.threat_b_18@a e.threat_b_19@a)

estat ic //AIC = , BIC =

*Calculate Posterior Probabilities

predict pr5_*, classposteriorpr

egen maxprob5 = rowmax (pr5_1 pr5_2 pr5_3 pr5_4 pr5_5)

gen class5 = 1 if pr5_1 == maxprob5

replace class5 = 2 if pr5_2 == maxprob5

replace class5 = 3 if pr5_3 == maxprob5

replace class5 = 4 if pr5_4 == maxprob5

replace class5 = 4 if pr5_4 == maxprob5

bysort class5: summ pr5_*

**#---Define Constraints---------------------------------------------------------

/* Within each class, we apply a series of constraints to ensure that the mean

change in threat belief remains consistent across all time points. We then test

whether the model performs better with these constraints or without them. */

constraint 1 _b[threat_b_3:1.C]-_b[threat_b_2:1.C]=_b[threat_b_2:1.C]-_b[threat_b_1:1.C]

constraint 2 _b[threat_b_4:1.C]-_b[threat_b_3:1.C]=_b[threat_b_3:1.C]-_b[threat_b_2:1.C]

constraint 3 _b[threat_b_5:1.C]-_b[threat_b_4:1.C]=_b[threat_b_4:1.C]-_b[threat_b_3:1.C]

constraint 4 _b[threat_b_6:1.C]-_b[threat_b_5:1.C]=_b[threat_b_5:1.C]-_b[threat_b_4:1.C]

constraint 5 _b[threat_b_7:1.C]-_b[threat_b_6:1.C]=_b[threat_b_6:1.C]-_b[threat_b_5:1.C]

constraint 6 _b[threat_b_8:1.C]-_b[threat_b_7:1.C]=_b[threat_b_7:1.C]-_b[threat_b_6:1.C]

constraint 7 _b[threat_b_9:1.C]-_b[threat_b_8:1.C]=_b[threat_b_8:1.C]-_b[threat_b_7:1.C]

constraint 8 _b[threat_b_10:1.C]-_b[threat_b_9:1.C]=_b[threat_b_9:1.C]-_b[threat_b_8:1.C]

constraint 9 _b[threat_b_11:1.C]-_b[threat_b_10:1.C]=_b[threat_b_10:1.C]-_b[threat_b_9:1.C]

constraint 10 _b[threat_b_12:1.C]-_b[threat_b_11:1.C]=_b[threat_b_11:1.C]-_b[threat_b_10:1.C]

constraint 11 _b[threat_b_13:1.C]-_b[threat_b_12:1.C]=_b[threat_b_12:1.C]-_b[threat_b_11:1.C]

constraint 12 _b[threat_b_14:1.C]-_b[threat_b_13:1.C]=_b[threat_b_13:1.C]-_b[threat_b_12:1.C]

constraint 13 _b[threat_b_15:1.C]-_b[threat_b_14:1.C]=_b[threat_b_14:1.C]-_b[threat_b_13:1.C]

constraint 14 _b[threat_b_16:1.C]-_b[threat_b_15:1.C]=_b[threat_b_15:1.C]-_b[threat_b_14:1.C]

constraint 15 _b[threat_b_17:1.C]-_b[threat_b_16:1.C]=_b[threat_b_16:1.C]-_b[threat_b_15:1.C]

constraint 16 _b[threat_b_18:1.C]-_b[threat_b_17:1.C]=_b[threat_b_17:1.C]-_b[threat_b_16:1.C]

constraint 17 _b[threat_b_19:1.C]-_b[threat_b_18:1.C]=_b[threat_b_18:1.C]-_b[threat_b_17:1.C]

constraint 18 _b[threat_b_3:2.C]-_b[threat_b_2:2.C]=_b[threat_b_2:2.C]-_b[threat_b_1:2.C]

constraint 19 _b[threat_b_4:2.C]-_b[threat_b_3:2.C]=_b[threat_b_3:2.C]-_b[threat_b_2:2.C]

constraint 20 _b[threat_b_5:2.C]-_b[threat_b_4:2.C]=_b[threat_b_4:2.C]-_b[threat_b_3:2.C]

constraint 21 _b[threat_b_6:2.C]-_b[threat_b_5:2.C]=_b[threat_b_5:2.C]-_b[threat_b_4:2.C]

constraint 22 _b[threat_b_7:2.C]-_b[threat_b_6:2.C]=_b[threat_b_6:2.C]-_b[threat_b_5:2.C]

constraint 23 _b[threat_b_8:2.C]-_b[threat_b_7:2.C]=_b[threat_b_7:2.C]-_b[threat_b_6:2.C]

constraint 24 _b[threat_b_9:2.C]-_b[threat_b_8:2.C]=_b[threat_b_8:2.C]-_b[threat_b_7:2.C]

constraint 25 _b[threat_b_10:2.C]-_b[threat_b_9:2.C]=_b[threat_b_9:2.C]-_b[threat_b_8:2.C]

constraint 26 _b[threat_b_11:2.C]-_b[threat_b_10:2.C]=_b[threat_b_10:2.C]-_b[threat_b_9:2.C]

constraint 27 _b[threat_b_12:2.C]-_b[threat_b_11:2.C]=_b[threat_b_11:2.C]-_b[threat_b_10:2.C]

constraint 28 _b[threat_b_13:2.C]-_b[threat_b_12:2.C]=_b[threat_b_12:2.C]-_b[threat_b_11:2.C]

constraint 29 _b[threat_b_14:2.C]-_b[threat_b_13:2.C]=_b[threat_b_13:2.C]-_b[threat_b_12:2.C]

constraint 30 _b[threat_b_15:2.C]-_b[threat_b_14:2.C]=_b[threat_b_14:2.C]-_b[threat_b_13:2.C]

constraint 31 _b[threat_b_16:2.C]-_b[threat_b_15:2.C]=_b[threat_b_15:2.C]-_b[threat_b_14:2.C]

constraint 32 _b[threat_b_17:2.C]-_b[threat_b_16:2.C]=_b[threat_b_16:2.C]-_b[threat_b_15:2.C]

constraint 33 _b[threat_b_18:2.C]-_b[threat_b_17:2.C]=_b[threat_b_17:2.C]-_b[threat_b_16:2.C]

constraint 34 _b[threat_b_19:2.C]-_b[threat_b_18:2.C]=_b[threat_b_18:2.C]-_b[threat_b_17:2.C]

constraint 35 _b[threat_b_3:3.C]-_b[threat_b_2:3.C]=_b[threat_b_2:3.C]-_b[threat_b_1:3.C]

constraint 36 _b[threat_b_4:3.C]-_b[threat_b_3:3.C]=_b[threat_b_3:3.C]-_b[threat_b_2:3.C]

constraint 37 _b[threat_b_5:3.C]-_b[threat_b_4:3.C]=_b[threat_b_4:3.C]-_b[threat_b_3:3.C]

constraint 38 _b[threat_b_6:3.C]-_b[threat_b_5:3.C]=_b[threat_b_5:3.C]-_b[threat_b_4:3.C]

constraint 39 _b[threat_b_7:3.C]-_b[threat_b_6:3.C]=_b[threat_b_6:3.C]-_b[threat_b_5:3.C]

constraint 40 _b[threat_b_8:3.C]-_b[threat_b_7:3.C]=_b[threat_b_7:3.C]-_b[threat_b_6:3.C]

constraint 41 _b[threat_b_9:3.C]-_b[threat_b_8:3.C]=_b[threat_b_8:3.C]-_b[threat_b_7:3.C]

constraint 42 _b[threat_b_10:3.C]-_b[threat_b_9:3.C]=_b[threat_b_9:3.C]-_b[threat_b_8:3.C]

constraint 43 _b[threat_b_11:3.C]-_b[threat_b_10:3.C]=_b[threat_b_10:3.C]-_b[threat_b_9:3.C]

constraint 44 _b[threat_b_12:3.C]-_b[threat_b_11:3.C]=_b[threat_b_11:3.C]-_b[threat_b_10:3.C]

constraint 45 _b[threat_b_13:3.C]-_b[threat_b_12:3.C]=_b[threat_b_12:3.C]-_b[threat_b_11:3.C]

constraint 46 _b[threat_b_14:3.C]-_b[threat_b_13:3.C]=_b[threat_b_13:3.C]-_b[threat_b_12:3.C]

constraint 47 _b[threat_b_15:3.C]-_b[threat_b_14:3.C]=_b[threat_b_14:3.C]-_b[threat_b_13:3.C]

constraint 48 _b[threat_b_16:3.C]-_b[threat_b_15:3.C]=_b[threat_b_15:3.C]-_b[threat_b_14:3.C]

constraint 49 _b[threat_b_17:3.C]-_b[threat_b_16:3.C]=_b[threat_b_16:3.C]-_b[threat_b_15:3.C]

constraint 50 _b[threat_b_18:3.C]-_b[threat_b_17:3.C]=_b[threat_b_17:3.C]-_b[threat_b_16:3.C]

constraint 51 _b[threat_b_19:3.C]-_b[threat_b_18:3.C]=_b[threat_b_18:3.C]-_b[threat_b_17:3.C]

constraint 52 _b[threat_b_3:4.C]-_b[threat_b_2:4.C]=_b[threat_b_2:4.C]-_b[threat_b_1:4.C]

constraint 53 _b[threat_b_4:4.C]-_b[threat_b_3:4.C]=_b[threat_b_3:4.C]-_b[threat_b_2:4.C]

constraint 54 _b[threat_b_5:4.C]-_b[threat_b_4:4.C]=_b[threat_b_4:4.C]-_b[threat_b_3:4.C]

constraint 55 _b[threat_b_6:4.C]-_b[threat_b_5:4.C]=_b[threat_b_5:4.C]-_b[threat_b_4:4.C]

constraint 56 _b[threat_b_7:4.C]-_b[threat_b_6:4.C]=_b[threat_b_6:4.C]-_b[threat_b_5:4.C]

constraint 57 _b[threat_b_8:4.C]-_b[threat_b_7:4.C]=_b[threat_b_7:4.C]-_b[threat_b_6:4.C]

constraint 58 _b[threat_b_9:4.C]-_b[threat_b_8:4.C]=_b[threat_b_8:4.C]-_b[threat_b_7:4.C]

constraint 59 _b[threat_b_10:4.C]-_b[threat_b_9:4.C]=_b[threat_b_9:4.C]-_b[threat_b_8:4.C]

constraint 60 _b[threat_b_11:4.C]-_b[threat_b_10:4.C]=_b[threat_b_10:4.C]-_b[threat_b_9:4.C]

constraint 61 _b[threat_b_12:4.C]-_b[threat_b_11:4.C]=_b[threat_b_11:4.C]-_b[threat_b_10:4.C]

constraint 62 _b[threat_b_13:4.C]-_b[threat_b_12:4.C]=_b[threat_b_12:4.C]-_b[threat_b_11:4.C]

constraint 63 _b[threat_b_14:4.C]-_b[threat_b_13:4.C]=_b[threat_b_13:4.C]-_b[threat_b_12:4.C]

constraint 64 _b[threat_b_15:4.C]-_b[threat_b_14:4.C]=_b[threat_b_14:4.C]-_b[threat_b_13:4.C]

constraint 65 _b[threat_b_16:4.C]-_b[threat_b_15:4.C]=_b[threat_b_15:4.C]-_b[threat_b_14:4.C]

constraint 66 _b[threat_b_17:4.C]-_b[threat_b_16:4.C]=_b[threat_b_16:4.C]-_b[threat_b_15:4.C]

constraint 67 _b[threat_b_18:4.C]-_b[threat_b_17:4.C]=_b[threat_b_17:4.C]-_b[threat_b_16:4.C]

constraint 68 _b[threat_b_19:4.C]-_b[threat_b_18:4.C]=_b[threat_b_18:4.C]-_b[threat_b_17:4.C]

**#---Comparison of Models -------------------------------------------------

/* Once we establish a class solution, we can explore three types of latent

trajectory models:

Model 1: Unconditional Trajectory Classes and Unconditional Class Probabilities

This basic model estimates unconditional class probabilities, allowing us to

examine class-covariate associations separately.

Model 2: Unconditional Trajectory Classes and Conditional Class Probabilities

In this model, class membership probabilities are conditioned on participant

covariates.

Model 3: Conditional Trajectory Classes and Unconditional Class Probabilities

Here, we condition on covariates before defining the classes.

We compare these models using the Bayesian Information Criteria, posterior

probabilities, and the plausibility of the resulting trajectory plots. */

**#---Model 1--------------------------------------------------------------------

/*Model 1 is an Unconditional Trajectory Classes and Unconditional Class

Probabilities Model. In this model, we specify the same structure as previously

described but include constraints to test whether they improve model fit. We

calculate posterior probabilities and, in our study, found that the inclusion of

constraints enhanced model fit. As a result, we incorporate these constraints into

all subsequent models. This approach allows us to plot the trajectories and

examine the associations between the identified classes and baseline predictors.*/

*Four Class Solution

gsem (threat_b_1 threat_b_2 threat_b_3 threat_b_4 threat_b_5 threat_b_6 threat_b_7 threat_b_8 threat_b_9 threat_b_10 threat_b_11 threat_b_12 threat_b_13 threat_b_14 threat_b_15 threat_b_16 threat_b_17 threat_b_18 threat_b_19<-), //

lclass (C 4) constraints (1 2 3 4 5 6 7 8 9 10 11 12 13 14 15 16 17 18 19 20 21 22 23 24 25 26 27 28 29 30 31 32 33 34 35 36 37 38 39 40 41 42 43 44 45 46 47 48 49 50 51 52 53 54 55 56 57 58 59 60 61 62 63 64 65 66 67 68) //

var(e.threat_b_1@a e.threat_b_2@a e.threat_b_3@a e.threat_b_4@a e.threat_b_5@a e.threat_b_6@a e.threat_b_7@a e.threat_b_8@a e.threat_b_9@a e.threat_b_10@a e.threat_b_11@a e.threat_b_12@a e.threat_b_13@a e.threat_b_14@a e.threat_b_15@a e.threat_b_16@a e.threat_b_17@a e.threat_b_18@a e.threat_b_19@a)

estat ic // AIC = , BIC =

*Calculate Posterior Probabilities

predict pr_*, classposteriorpr

egen maxprob1 = rowmax(pr_1 pr_2 pr_3 pr_4)

gen class1 = 1 if pr_1 == maxprob1

replace class1 = 2 if pr_2 == maxprob1

replace class1 = 3 if pr_3 == maxprob1

replace class1 = 4 if pr_4 == maxprob1

bysort class1: sum pr_*

*Plot of Class trajectories

profileplot threat_b_1 threat_b_2 threat_b_3 threat_b_4 threat_b_5 threat_b_6 threat_b_7 threat_b_8 threat_b_9 threat_b_10 threat_b_11 threat_b_12 threat_b_13 threat_b_14 threat_b_15 threat_b_16 threat_b_17 threat_b_18 threat_b_19,//

by(class1) name(model_1, replace)

*Association between class and covariates

capture program drop predictors

program predictors

syntax varlist

foreach var of local varlist {

mlogit class1 `var', baseoutcome(4) //baseoutcome = largest class

}

end

predictors age employment living_situation gender marital_status conviction psyrats_total wemwbs rgpts...

/*Specify a list of all baseline covarites that may be associated with class

membership. After this code, examine the relationship between covariates and

class. Include all variables associated with class into an adjusted model.*/

*Adjusted association between class and covariates

mlogit class1 conviction rgpts_ptb_total expectancy bcss_others_positive_total vulnerability, baseoutcome(4)

/*Here you can refine the model, the ensure that each predictor is associated with

class, when adjusting for other predictors. */

*Final Refined Model - Adjusted association between class and covariates

mlogit class1 conviction expectancy bcss_others_positive_total, baseoutcome(4)

**#---Model 2--------------------------------------------------------------------

/* Model 2 is an Unconditional Trajectory Classes and Conditional Class

Probabilities Model. In this approach, we incorporate covariates from our refined

multinomial logistic regression model to predict class membership within the

model. Multiple versions of Model 2 can be compared, each with different covariates

predicting class membership. The final model should be selected based on Bayesian

Information Criteria (BIC), posterior probabilities, and theoretical plausibility.

In our analysis, we compared two versions: Model 2a and Model 2b, each using

covariates from the multinomial models outlined earlier. We concluded that Model

2b provided a better fit to the data than Model 2a, whilst also being more

parsimonious.*/

// Model 2a - Five Predictors of Class

gsem (threat_b_1 threat_b_2 threat_b_3 threat_b_4 threat_b_5 threat_b_6 threat_b_7 threat_b_8 threat_b_9 threat_b_10 threat_b_11 threat_b_12 threat_b_13 threat_b_14 threat_b_15 threat_b_16 threat_b_17 threat_b_18 threat_b_19<-) ///

(C <- conviction rgpts_ptb_total expectancy bcss_others_positive_total vulnerability), ///

lclass (C 4) constraints (1 2 3 4 5 6 7 8 9 10 11 12 13 14 15 16 17 18 19 20 21 22 23 24 25 26 27 28 29 30 31 32 33 34 35 36 37 38 39 40 41 42 43 44 45 46 47 48 49 50 51 52 53 54 55 56 57 58 59 60 61 62 63 64 65 66 67 68) ///

var(e.threat_b_1@a e.threat_b_2@a e.threat_b_3@a e.threat_b_4@a e.threat_b_5@a e.threat_b_6@a e.threat_b_7@a e.threat_b_8@a e.threat_b_9@a e.threat_b_10@a e.threat_b_11@a e.threat_b_12@a e.threat_b_13@a e.threat_b_14@a e.threat_b_15@a e.threat_b_16@a e.threat_b_17@a e.threat_b_18@a e.threat_b_19@a)

estat ic //BIC =

//Posterior Probabilities

predict pr2a_*, classposteriorpr

egen maxprob2a = rowmax(pr2a_1 pr2a_2 pr2a_3 pr2a_4)

gen class2a = 1 if pr2a_1 == maxprob2a

replace class2a = 2 if pr2a_2 == maxprob2a

replace class2a = 3 if pr2a_3 == maxprob2a

replace class2a = 4 if pr2a_4 == maxprob2a

bysort class2a: sum pr2a_*

//Plot of Class Trajectories

profileplot threat_b_1 threat_b_2 threat_b_3 threat_b_4 threat_b_5 threat_b_6 threat_b_7 threat_b_8 threat_b_9 threat_b_10 threat_b_11 threat_b_12 threat_b_13 threat_b_14 threat_b_15 threat_b_16 threat_b_17 threat_b_18 threat_b_19, by(class2a) name(model2a, replace)

// Model 2b - Three Predictors of Class

gsem (threat_b_1 threat_b_2 threat_b_3 threat_b_4 threat_b_5 threat_b_6 threat_b_7 threat_b_8 threat_b_9 threat_b_10 threat_b_11 threat_b_12 threat_b_13 threat_b_14 threat_b_15 threat_b_16 threat_b_17 threat_b_18 threat_b_19<-) ///

(C <- conviction expectancy bcss_others_positive_total), lclass (C 4) ///

constraints (1 2 3 4 5 6 7 8 9 10 11 12 13 14 15 16 17 18 19 20 21 22 23 24 25 26 27 28 29 30 31 32 33 34 35 36 37 38 39 40 41 42 43 44 45 46 47 48 49 50 51 52 53 54 55 56 57 58 59 60 61 62 63 64 65 66 67 68) ///

var(e.threat_b_1@a e.threat_b_2@a e.threat_b_3@a e.threat_b_4@a e.threat_b_5@a e.threat_b_6@a e.threat_b_7@a e.threat_b_8@a e.threat_b_9@a e.threat_b_10@a e.threat_b_11@a e.threat_b_12@a e.threat_b_13@a e.threat_b_14@a e.threat_b_15@a e.threat_b_16@a e.threat_b_17@a e.threat_b_18@a e.threat_b_19@a)

estat ic //BIC =

//Posterior Probabilities

predict pr2b_*, classposteriorpr

egen maxprob2b = rowmax(pr2b_1 pr2b_2 pr2b_3 pr2b_4)

gen class2b = 1 if pr2b_1 == maxprob2b

replace class2b = 2 if pr2b_2 == maxprob2b

replace class2b = 3 if pr2b_3 == maxprob2b

replace class2b = 4 if pr2b_4 == maxprob2b

bysort class2b: sum pr2b_*

//Plot of Class Trajectories

profileplot threat_b_1 threat_b_2 threat_b_3 threat_b_4 threat_b_5 threat_b_6 threat_b_7 threat_b_8 threat_b_9 threat_b_10 threat_b_11 threat_b_12 threat_b_13 threat_b_14 threat_b_15 threat_b_16 threat_b_17 threat_b_18 threat_b_19, by(class2b) name(model2b, replace)

**#---Model 3--------------------------------------------------------------------

/*Model 3 is a Conditional Trajectory Classes and Unonditional Class Probabilities

Model. In this model, covariates from our refined multinomial logistic regression

model are used to predict the outcome. This is achieved by defining constraints

based on the final predictors of class and incorporating these constraints within

the model. It is important to note that this model may encounter convergence

issues, which should be carefully monitored during the analysis.*/

//Model 3

gsem ///

(threat_b_1 <- conviction@b expectancy@c bcss_others_positive_total@d) ///

(threat_b_2 <- conviction@2*b expectancy@2*c bcss_others_positive_total@2*d) ///

(threat_b_3 <- conviction@3*b expectancy@3*c bcss_others_positive_total@3*d) ///

(threat_b_4 <- conviction@4*b expectancy@4*c bcss_others_positive_total@4*d) ///

(threat_b_5 <- conviction@5*b expectancy@5*c bcss_others_positive_total@5*d) ///

(threat_b_6 <- conviction@6*b expectancy@6*c bcss_others_positive_total@6*d) ///

(threat_b_7 <- conviction@7*b expectancy@7*c bcss_others_positive_total@7*d) ///

(threat_b_8 <- conviction@8*b expectancy@8*c bcss_others_positive_total@8*d) ///

(threat_b_9 <- conviction@9*b expectancy@9*c bcss_others_positive_total@9*d) ///

(threat_b_10 <- conviction@10*b expectancy@10*c bcss_others_positive_total@10*d) ///

(threat_b_11 <- conviction@11*b expectancy@11*c bcss_others_positive_total@11*d) ///

(threat_b_12 <- conviction@12*b expectancy@12*c bcss_others_positive_total@12*d) ///

(threat_b_13 <- conviction@13*b expectancy@13*c bcss_others_positive_total@13*d) ///

(threat_b_14 <- conviction@14*b expectancy@14*c bcss_others_positive_total@14*d) ///

(threat_b_15 <- conviction@15*b expectancy@15*c bcss_others_positive_total@15*d) ///

(threat_b_16 <- conviction@16*b expectancy@16*c bcss_others_positive_total@16*d) ///

(threat_b_17 <- conviction@17*b expectancy@17*c bcss_others_positive_total@17*d) ///

(threat_b_18 <- conviction@18*b expectancy@18*c bcss_others_positive_total@18*d) ///

(threat_b_19 <- conviction@19*b expectancy@19*c bcss_others_positive_total@19*d), ///

lclass (C 4) constraints (1 2 3 4 5 6 7 8 9 10 11 12 13 14 15 16 17 18 19 20 21 22 23 24 25 26 27 28 29 30 31 32 33 34 35 36 37 38 39 40 41 42 43 44 45 46 47 48 49 50 51 52 53 54 55 56 57 58 59 60 61 62 63 64 65 66 67 68) ///

var(e.threat_b_1@a e.threat_b_2@a e.threat_b_3@a e.threat_b_4@a e.threat_b_5@a e.threat_b_6@a e.threat_b_7@a e.threat_b_8@a e.threat_b_9@a e.threat_b_10@a e.threat_b_11@a e.threat_b_12@a e.threat_b_13@a e.threat_b_14@a e.threat_b_15@a e.threat_b_16@a e.threat_b_17@a e.threat_b_18@a e.threat_b_19@a)

estat ic //BIC =

//Posterior Probabilities

predict pr3b_*, classposteriorpr

egen maxprob3b = rowmax(pr3b_1 pr3b_2 pr3b_3 pr3b_4)

gen class3 = 1 if pr3b_1 == maxprob3b

replace class3 = 2 if pr3b_2 == maxprob3b

replace class3 = 3 if pr3b_3 == maxprob3b

replace class3 = 4 if pr3b_4 == maxprob3b

bysort class3: sum pr3b_*

//Plot of Class trajectories

profileplot threat_b_1 threat_b_2 threat_b_3 threat_b_4 threat_b_5 threat_b_6 threat_b_7 threat_b_8 threat_b_9 threat_b_10 threat_b_11 threat_b_12 threat_b_13 threat_b_14 threat_b_15 threat_b_16 threat_b_17 threat_b_18 threat_b_19, by(class3) name(model3, replace)

**#---Final Model Plots----------------------------------------------------------

//We found that the optimal latent class model was Model 2b.

//Plot of Class Trajectories For Final Model

profileplot threat_b_1 threat_b_2 threat_b_3 threat_b_4 threat_b_5 threat_b_6 threat_b_7 threat_b_8 threat_b_9 threat_b_10 threat_b_11 threat_b_12 threat_b_13 threat_b_14 threat_b_15 threat_b_16 threat_b_17 threat_b_18 threat_b_19, by(class2b) name(model2b, replace)

//We can plot the independent conviction score at baseline, 6 months and 12 months by class

preserve

rename conviction conviction1

rename conviction_6m_primaryoutcome conviction2

rename conviction_12m_primaryoutcome conviction3

reshape long conviction, i(participant_id) j(time)

collapse conviction, by(class2b time)

twoway (line conviction time if class2b ==1) (line conviction time if class2b ==2) (line conviction time if class2b ==3) (line conviction time if class2b ==4), legend(order(1 "Class 1" 2 "Class 2" 3 "Class 3" 4 "Class 4")) ytitle("mean conviction score") xtitle("Time")

restore

**---End of Analysis------------------------------------------------------------

1. **Principal Trajectories Analysis**

The following code was used to conduct a principal trajectories analysis

following the model presented^2^ within Dunn G, Emsley R, Liu H, et al. Evaluation

and validation of social and psychological markers in randomised trials of complex

interventions in mental health: a methodological research programme. Health

Technology Assessment (Winchester, England). 2015 Nov;19(93):1-115, v-vi.

DOI: 10.3310/hta19930.

This script implements a principal trajectories model. First, we fit the optimal

latent trajectory model to our data, following the methodology outlined in Jenner

et al., 2024^1^. Using the posterior probabilities, we estimate class membership for

participants in the treatment arm. Using the predicted probabilities, we estimate

class membership for participants in the control arm. This approach enables us to

assign control arm participants to the latent class they would likely belong to if

they had been allocated to the Feeling Safe treatment.

We then estimate the intention-to-treat effect within each class. To explore

potential mediators further, we calculate the 'a' path within each class to screen

for mechanisms by which the Feeling Safe intervention operates.

**Contents of Script**

- Data Preparation

- Fit Latent Class Trajectory Model

- Assign Intervention Arm

- Assign Control Arm

- Estimate ITT Effect

- Explore Potential Mediators

**#---Data Preparation-----------------------------------------------------------

clear

cd "/DataFileLocation" //Set the directory to the location of your file

use FeelingSafeData //Open Data file

**#---Fit Latent Class Trajectory Model ------------------------------

/* Within each class, we apply a series of constraints to ensure that the mean

change in threat belief remains consistent across all time points. */

constraint 1 _b[threat_b_3:1.C]-_b[threat_b_2:1.C]=_b[threat_b_2:1.C]-_b[threat_b_1:1.C]

constraint 2 _b[threat_b_4:1.C]-_b[threat_b_3:1.C]=_b[threat_b_3:1.C]-_b[threat_b_2:1.C]

constraint 3 _b[threat_b_5:1.C]-_b[threat_b_4:1.C]=_b[threat_b_4:1.C]-_b[threat_b_3:1.C]

constraint 4 _b[threat_b_6:1.C]-_b[threat_b_5:1.C]=_b[threat_b_5:1.C]-_b[threat_b_4:1.C]

constraint 5 _b[threat_b_7:1.C]-_b[threat_b_6:1.C]=_b[threat_b_6:1.C]-_b[threat_b_5:1.C]

constraint 6 _b[threat_b_8:1.C]-_b[threat_b_7:1.C]=_b[threat_b_7:1.C]-_b[threat_b_6:1.C]

constraint 7 _b[threat_b_9:1.C]-_b[threat_b_8:1.C]=_b[threat_b_8:1.C]-_b[threat_b_7:1.C]

constraint 8 _b[threat_b_10:1.C]-_b[threat_b_9:1.C]=_b[threat_b_9:1.C]-_b[threat_b_8:1.C]

constraint 9 _b[threat_b_11:1.C]-_b[threat_b_10:1.C]=_b[threat_b_10:1.C]-_b[threat_b_9:1.C]

constraint 10 _b[threat_b_12:1.C]-_b[threat_b_11:1.C]=_b[threat_b_11:1.C]-_b[threat_b_10:1.C]

constraint 11 _b[threat_b_13:1.C]-_b[threat_b_12:1.C]=_b[threat_b_12:1.C]-_b[threat_b_11:1.C]

constraint 12 _b[threat_b_14:1.C]-_b[threat_b_13:1.C]=_b[threat_b_13:1.C]-_b[threat_b_12:1.C]

constraint 13 _b[threat_b_15:1.C]-_b[threat_b_14:1.C]=_b[threat_b_14:1.C]-_b[threat_b_13:1.C]

constraint 14 _b[threat_b_16:1.C]-_b[threat_b_15:1.C]=_b[threat_b_15:1.C]-_b[threat_b_14:1.C]

constraint 15 _b[threat_b_17:1.C]-_b[threat_b_16:1.C]=_b[threat_b_16:1.C]-_b[threat_b_15:1.C]

constraint 16 _b[threat_b_18:1.C]-_b[threat_b_17:1.C]=_b[threat_b_17:1.C]-_b[threat_b_16:1.C]

constraint 17 _b[threat_b_19:1.C]-_b[threat_b_18:1.C]=_b[threat_b_18:1.C]-_b[threat_b_17:1.C]

constraint 18 _b[threat_b_3:2.C]-_b[threat_b_2:2.C]=_b[threat_b_2:2.C]-_b[threat_b_1:2.C]

constraint 19 _b[threat_b_4:2.C]-_b[threat_b_3:2.C]=_b[threat_b_3:2.C]-_b[threat_b_2:2.C]

constraint 20 _b[threat_b_5:2.C]-_b[threat_b_4:2.C]=_b[threat_b_4:2.C]-_b[threat_b_3:2.C]

constraint 21 _b[threat_b_6:2.C]-_b[threat_b_5:2.C]=_b[threat_b_5:2.C]-_b[threat_b_4:2.C]

constraint 22 _b[threat_b_7:2.C]-_b[threat_b_6:2.C]=_b[threat_b_6:2.C]-_b[threat_b_5:2.C]

constraint 23 _b[threat_b_8:2.C]-_b[threat_b_7:2.C]=_b[threat_b_7:2.C]-_b[threat_b_6:2.C]

constraint 24 _b[threat_b_9:2.C]-_b[threat_b_8:2.C]=_b[threat_b_8:2.C]-_b[threat_b_7:2.C]

constraint 25 _b[threat_b_10:2.C]-_b[threat_b_9:2.C]=_b[threat_b_9:2.C]-_b[threat_b_8:2.C]

constraint 26 _b[threat_b_11:2.C]-_b[threat_b_10:2.C]=_b[threat_b_10:2.C]-_b[threat_b_9:2.C]

constraint 27 _b[threat_b_12:2.C]-_b[threat_b_11:2.C]=_b[threat_b_11:2.C]-_b[threat_b_10:2.C]

constraint 28 _b[threat_b_13:2.C]-_b[threat_b_12:2.C]=_b[threat_b_12:2.C]-_b[threat_b_11:2.C]

constraint 29 _b[threat_b_14:2.C]-_b[threat_b_13:2.C]=_b[threat_b_13:2.C]-_b[threat_b_12:2.C]

constraint 30 _b[threat_b_15:2.C]-_b[threat_b_14:2.C]=_b[threat_b_14:2.C]-_b[threat_b_13:2.C]

constraint 31 _b[threat_b_16:2.C]-_b[threat_b_15:2.C]=_b[threat_b_15:2.C]-_b[threat_b_14:2.C]

constraint 32 _b[threat_b_17:2.C]-_b[threat_b_16:2.C]=_b[threat_b_16:2.C]-_b[threat_b_15:2.C]

constraint 33 _b[threat_b_18:2.C]-_b[threat_b_17:2.C]=_b[threat_b_17:2.C]-_b[threat_b_16:2.C]

constraint 34 _b[threat_b_19:2.C]-_b[threat_b_18:2.C]=_b[threat_b_18:2.C]-_b[threat_b_17:2.C]

constraint 35 _b[threat_b_3:3.C]-_b[threat_b_2:3.C]=_b[threat_b_2:3.C]-_b[threat_b_1:3.C]

constraint 36 _b[threat_b_4:3.C]-_b[threat_b_3:3.C]=_b[threat_b_3:3.C]-_b[threat_b_2:3.C]

constraint 37 _b[threat_b_5:3.C]-_b[threat_b_4:3.C]=_b[threat_b_4:3.C]-_b[threat_b_3:3.C]

constraint 38 _b[threat_b_6:3.C]-_b[threat_b_5:3.C]=_b[threat_b_5:3.C]-_b[threat_b_4:3.C]

constraint 39 _b[threat_b_7:3.C]-_b[threat_b_6:3.C]=_b[threat_b_6:3.C]-_b[threat_b_5:3.C]

constraint 40 _b[threat_b_8:3.C]-_b[threat_b_7:3.C]=_b[threat_b_7:3.C]-_b[threat_b_6:3.C]

constraint 41 _b[threat_b_9:3.C]-_b[threat_b_8:3.C]=_b[threat_b_8:3.C]-_b[threat_b_7:3.C]

constraint 42 _b[threat_b_10:3.C]-_b[threat_b_9:3.C]=_b[threat_b_9:3.C]-_b[threat_b_8:3.C]

constraint 43 _b[threat_b_11:3.C]-_b[threat_b_10:3.C]=_b[threat_b_10:3.C]-_b[threat_b_9:3.C]

constraint 44 _b[threat_b_12:3.C]-_b[threat_b_11:3.C]=_b[threat_b_11:3.C]-_b[threat_b_10:3.C]

constraint 45 _b[threat_b_13:3.C]-_b[threat_b_12:3.C]=_b[threat_b_12:3.C]-_b[threat_b_11:3.C]

constraint 46 _b[threat_b_14:3.C]-_b[threat_b_13:3.C]=_b[threat_b_13:3.C]-_b[threat_b_12:3.C]

constraint 47 _b[threat_b_15:3.C]-_b[threat_b_14:3.C]=_b[threat_b_14:3.C]-_b[threat_b_13:3.C]

constraint 48 _b[threat_b_16:3.C]-_b[threat_b_15:3.C]=_b[threat_b_15:3.C]-_b[threat_b_14:3.C]

constraint 49 _b[threat_b_17:3.C]-_b[threat_b_16:3.C]=_b[threat_b_16:3.C]-_b[threat_b_15:3.C]

constraint 50 _b[threat_b_18:3.C]-_b[threat_b_17:3.C]=_b[threat_b_17:3.C]-_b[threat_b_16:3.C]

constraint 51 _b[threat_b_19:3.C]-_b[threat_b_18:3.C]=_b[threat_b_18:3.C]-_b[threat_b_17:3.C]

constraint 52 _b[threat_b_3:4.C]-_b[threat_b_2:4.C]=_b[threat_b_2:4.C]-_b[threat_b_1:4.C]

constraint 53 _b[threat_b_4:4.C]-_b[threat_b_3:4.C]=_b[threat_b_3:4.C]-_b[threat_b_2:4.C]

constraint 54 _b[threat_b_5:4.C]-_b[threat_b_4:4.C]=_b[threat_b_4:4.C]-_b[threat_b_3:4.C]

constraint 55 _b[threat_b_6:4.C]-_b[threat_b_5:4.C]=_b[threat_b_5:4.C]-_b[threat_b_4:4.C]

constraint 56 _b[threat_b_7:4.C]-_b[threat_b_6:4.C]=_b[threat_b_6:4.C]-_b[threat_b_5:4.C]

constraint 57 _b[threat_b_8:4.C]-_b[threat_b_7:4.C]=_b[threat_b_7:4.C]-_b[threat_b_6:4.C]

constraint 58 _b[threat_b_9:4.C]-_b[threat_b_8:4.C]=_b[threat_b_8:4.C]-_b[threat_b_7:4.C]

constraint 59 _b[threat_b_10:4.C]-_b[threat_b_9:4.C]=_b[threat_b_9:4.C]-_b[threat_b_8:4.C]

constraint 60 _b[threat_b_11:4.C]-_b[threat_b_10:4.C]=_b[threat_b_10:4.C]-_b[threat_b_9:4.C]

constraint 61 _b[threat_b_12:4.C]-_b[threat_b_11:4.C]=_b[threat_b_11:4.C]-_b[threat_b_10:4.C]

constraint 62 _b[threat_b_13:4.C]-_b[threat_b_12:4.C]=_b[threat_b_12:4.C]-_b[threat_b_11:4.C]

constraint 63 _b[threat_b_14:4.C]-_b[threat_b_13:4.C]=_b[threat_b_13:4.C]-_b[threat_b_12:4.C]

constraint 64 _b[threat_b_15:4.C]-_b[threat_b_14:4.C]=_b[threat_b_14:4.C]-_b[threat_b_13:4.C]

constraint 65 _b[threat_b_16:4.C]-_b[threat_b_15:4.C]=_b[threat_b_15:4.C]-_b[threat_b_14:4.C]

constraint 66 _b[threat_b_17:4.C]-_b[threat_b_16:4.C]=_b[threat_b_16:4.C]-_b[threat_b_15:4.C]

constraint 67 _b[threat_b_18:4.C]-_b[threat_b_17:4.C]=_b[threat_b_17:4.C]-_b[threat_b_16:4.C]

constraint 68 _b[threat_b_19:4.C]-_b[threat_b_18:4.C]=_b[threat_b_18:4.C]-_b[threat_b_17:4.C]

/*We specify our latent trajectory model, identified as the best fit to our data

in prior analyses. This is an unconditional trajectories and conditional class

probabilities model, where predictors influence class membership, and class

membership explains the outcome.

The model includes four latent classes. We impose constraints on the mean changes

of the outcome within classes and ensure that the residual error variance is

consistent across all measures of the outcome at each time point. */

// Latent Trajectory Model

gsem (threat_b_1 threat_b_2 threat_b_3 threat_b_4 threat_b_5 threat_b_6 threat_b_7 threat_b_8 threat_b_9 threat_b_10 threat_b_11 threat_b_12 threat_b_13 threat_b_14 threat_b_15 threat_b_16 threat_b_17 threat_b_18 threat_b_19<-) ///

(C <- conviction expectancy bcss_others_positive_total), lclass (C 4) ///

constraints (1 2 3 4 5 6 7 8 9 10 11 12 13 14 15 16 17 18 19 20 21 22 23 24 25 26 27 28 29 30 31 32 33 34 35 36 37 38 39 40 41 42 43 44 45 46 47 48 49 50 51 52 53 54 55 56 57 58 59 60 61 62 63 64 65 66 67 68) ///

var(e.threat_b_1@a e.threat_b_2@a e.threat_b_3@a e.threat_b_4@a e.threat_b_5@a e.threat_b_6@a e.threat_b_7@a e.threat_b_8@a e.threat_b_9@a e.threat_b_10@a e.threat_b_11@a e.threat_b_12@a e.threat_b_13@a e.threat_b_14@a e.threat_b_15@a e.threat_b_16@a e.threat_b_17@a e.threat_b_18@a e.threat_b_19@a)

estat ic //BIC =

**Note: For the next stages, we require no missing data in the predictors of

**trajectory class. We drop all participants with missing baseline data.

drop if conviction == . | expectancy == . | bcss_others_positive_total == .

**#---Assign Intervention Arm----------------------------------------

/*We calculate the posterior probabilities from the latent trajectory analysis and

assign all Feeling Safe participants to their most likely class. */

if allocation == 1 {

//Predict posterior probabilities for sample

predict pr_*, classposteriorpr

//Assign individuals in group 1 to their most likely class

egen maxprob = rowmax(pr_1 pr_2 pr_3 pr_4)

gen classall = .

replace classall = 2 if allocation == 1 & pr_1 == maxprob

replace classall = 1 if allocation == 1 & pr_2 == maxprob

replace classall = 4 if allocation == 1 & pr_3 == maxprob

replace classall = 3 if allocation == 1 & pr_4 == maxprob

}

**#---Assign Control Arm----------------------------------------

/*We then estimate the predicted probabilities, using the regression model within

the latent trajectories analysis and assign the control arm to the latent class

that they would most likely be in, if they had received Feeling Safe treatment.*/

//Estimate predicted probabilities for sample

predict pr2_*, classpr

//Assign individuals to their most likely class.

egen maxprob2 = rowmax(pr2_1 pr2_2 pr2_3 pr2_4)

gen classall2 = .

replace classall2 = 2 if allocation == 0 & pr2_1 == maxprob2

replace classall2 = 1 if allocation == 0 & pr2_2 == maxprob2

replace classall2 = 4 if allocation == 0 & pr2_3 == maxprob2

replace classall2 = 3 if allocation == 0 & pr2_4 == maxprob2

//We create a final class variable based off our previous assignments

gen class_final = .

replace class_final = classall if allocation == 1

replace class_final = classall2 if allocation == 0

*This code is creating a new variable for class membership, based on the

*posterior probabilities for the treatment arm assignment and the predicted

*probabilities for the control arm assigment.

//We assess the probabilities in our model

bysort classall (pr_*): summarize pr_*

bysort classall2 (pr2_*): summarize pr2_*

bysort class_final (maxprob2): summarize maxprob2

**#---Estimate ITT Effect------------------------------------------

/*We can now examine the effect of randomisation on the outcome within each class

separately. We consider the primary outcome at 6 and 12 months*/

//ITT Effect Final (6 Months)

regress conviction_6 conviction allocation

//ITT Effect Within Each Class

forval i = 1/4{

di "Effect of treatment on outcome within class `i'"

regress conviction_6 conviction allocation if class_final == `i'

}

//ITT Effect Final (12 Months)

regress conviction_12 conviction allocation

//ITT Effect Within Each Class

forval i = 1/4{

di "Effect of treatment on outcome within class `i'"

regress conviction_12 conviction allocation if class_final == `i'

}

**#---Explore Potential Mediators------------------------------------------

/* This is an exploratory mediation analysis. In the main Feeling Safe trial,

various potential mediators were examined, with some identified as significant

mediators. In this analysis, we aim to investigate how randomization is associated

with potential mediators within each class. The association between treatment and

mediator is assessed through the estimation of the 'a' path. If an association is

found, this suggests that the variable may serve as a mediator. Although further

analysis to calculate the indirect effect would typically follow, since the

relationship between mediator and outcome has already been established in the

Feeling Safe trial, we focus solely on the 'a' path as part of a screening process

for identifying potential mediators within each class. */

//Jumping to Conclusions

forval i = 1/4{

di "Effect of treatment on mediator within class `i'"

regress jtc_beads_6m allocation jtc_beads conviction if class_final == `i'

}

//Safety Behaviours

forval i = 1/4{

di "Effect of treatment on mediator within class `i'"

regress safety_behaviours_totalfreq_6m allocation safety_behaviours_totalfreq conviction if class_final == `i'

}

//Safety

forval i = 1/4{

di "Effect of treatment on mediator within class `i'"

regress safety_6m allocation safety conviction if class_final == `i'

}

//ISI

forval i = 1/4{

di "Effect of treatment on mediator within class `i'"

regress isi_total_6m allocation isi_total conviction if class_final == `i'

}

// PSWQ

forval i = 1/4{

di "Effect of treatment on mediator within class `i'"

regress pswq_total_6m allocation pswq_total conviction if class_final == `i'

}

//BCSS Self Neg

forval i = 1/4{

di "Effect of treatment on mediator within class `i'"

regress bcss_self_negative_total_6m allocation bcss_self_negative_total conviction if class_final == `i'

}

//BCSS Self Pos

forval i = 1/4{

di "Effect of treatment on mediator within class `i'"

regress bcss_self_positive_total_6m allocation bcss_self_positive_total conviction if class_final == `i'

}

//BCSS Others Neg

forval i = 1/4{

di "Effect of treatment on mediator within class `i'"

regress bcss_others_negative_total_6m allocation bcss_others_negative_total conviction if class_final == `i'

}

//BCSS others Pos

forval i = 1/4{

di "Effect of treatment on mediator within class `i'"

regress bcss_others_positive_total_6m allocation bcss_others_positive_total conviction if class_final == `i'

}

//Anomalous exp

forval i = 1/4{

di "Effect of treatment on mediator within class `i'"

regress anomalousexperiences_total_6m allocation anomalousexperiences_total conviction if class_final == `i'

}

//Vulnerability

forval i = 1/4{

di "Effect of treatment on mediator within class `i'"

regress vulnerability_6m allocation vulnerability conviction if class_final == `i'

}

//Belief Flexibilty - mads

forval i = 1/4{

di "Effect of treatment on mediator within class `i'"

regress mads_6m allocation mads conviction if class_final == `i'

}

**---End of Analysis------------------------------------------------------------

**References**

1. Jenner L, Payne M, Waite F, et al. Theory driven psychological therapy for persecutory delusions: trajectories of patient outcomes. *Psychol Med*. Published online November 18, 2024:1-9. doi:10.1017/S0033291724002113

2. Dunn G, Emsley R, Liu H, et al. Evaluation and validation of social and psychological markers in randomised trials of complex interventions in mental health: a methodological research programme. *Health Technol Assess (Rockv)*. 2015;19(93):1-116. doi:10.3310/hta19930
